# Direct Measurement of Crossover and Interfacial Resistance of Ion-Exchange Membranes in All-Vanadium Redox Flow Batteries

**DOI:** 10.3390/membranes10060126

**Published:** 2020-06-18

**Authors:** Yasser Ashraf Gandomi, Doug S. Aaron, Zachary B. Nolan, Arya Ahmadi, Matthew M. Mench

**Affiliations:** 1 Department of Mechanical, Aerospace and Biomedical Engineering, University of Tennessee, Knoxville, TN 37996, USA; ygandomi@mit.edu (Y.A.G.); daaron@utk.edu (D.S.A.); znolan1@vols.utk.edu (Z.B.N.); aahmadi2@vols.utk.edu (A.A.); 2 Department of Chemical Engineering, Massachusetts Institute of Technology, Cambridge, MA 02139, USA; 3 Energy and Transportation Science Division, Oak Ridge National Laboratory, Oak Ridge, TN 37831, USA

**Keywords:** redox flow batteries, membranes, crossover, interfacial impedance, ionic conductivity, UV/Vis spectroscopy

## Abstract

Among various components commonly used in redox flow batteries (RFBs), the separator plays a significant role, influencing resistance to current as well as capacity decay via unintended crossover. It is well-established that the ohmic overpotential is dominated by the membrane and interfacial resistance in most aqueous RFBs. The ultimate goal of engineering membranes is to improve the ionic conductivity while keeping crossover at a minimum. One of the major issues yet to be addressed is the contribution of interfacial phenomena in the influence of ionic and water transport through the membrane. In this work, we have utilized a novel experimental system capable of measuring the ionic crossover in real-time to quantify the permeability of ionic species. Specifically, we have focused on quantifying the contributions from the interfacial resistance to ionic crossover. The trade-off between the mass and ionic transport impedance caused by the interface of the membranes has been addressed. The MacMullin number has been quantified for a series of electrolyte configurations and a correlation between the ionic conductivity of the contacting electrolyte and the Nafion^®^ membrane has been established. The performance of individual ion-exchange membranes along with a stack of various separators have been explored. We have found that utilizing a stack of membranes is significantly beneficial in reducing the electroactive species crossover in redox flow batteries compared to a single membrane of the same fold thickness. For example, we have demonstrated that the utilization of five layers of Nafion^®^ 211 membrane reduces the crossover by 37% while only increasing the area-specific resistance (ASR) by 15% compared to a single layer Nafion^®^ 115 membrane. Therefore, the influence of interfacial impedance in reducing the vanadium ion crossover is substantially higher compared to a corresponding increase in ASR, indicating that mass and ohmic interfacial resistances are dissimilar. We have expanded our analysis to a combination of commercially available ion-exchange membranes and provided a design chart for membrane selection based on the application of interest (short duration/high-performance vs. long-term durability). The results of this study provide a deeper insight into the optimization of all-vanadium redox flow batteries (VRFBs).

## 1. Introduction

To fully integrate the renewable energy sources within the energy cycle (i.e., supply, distribution, and consumption), reliable energy storage technologies are needed [1,2]. Redox flow batteries (RFBs) store energy in flowable electrolytes or in a suspension of redox-active compounds in external reservoirs. RFBs enable decoupling energy storage from the power-generating reactor and allow design for the desired energy storage capacity and/or power rating of the particular application [2,3,4,5].

Since the electrodes and electrolytes are separated in RFBs, the electrolytes (low-potential and high-potential sides) are pumped through the reactor where they are separated by a separator or an ion-exchange membrane [6]. For RFBs utilizing transition metal salts as the electroactive redox compounds (e.g., all-vanadium redox flow batteries (VRFBs)), ion-exchange membranes (IEMs) are the leading choice for separating the low-potential and high-potential electrolytes. IEMs must provide a conductive ionic pathway for the charge balancing ions, be durable, have near zero electronic conductivity, and be preferentially selective in terms of mass transport [7,8,9,10,11,12,13].

Polymeric membranes (e.g., Nafion^®^ by DuPont^TM^) have widely been used for VRFBs, a recent review paper has summarized the application of various membranes for aqueous RFBs [14]. Nafion^®^ has been extensively utilized for VRFBs due to excellent durability and conductivity in acidic media. However, the selectivity of Nafion^®^ must be further improved for long-term applications, since vanadium ion transport through Nafion^®^ adversely affects the capacity. The undesired transport of vanadium ions across the membrane (i.e., crossover) results in self-discharge and reversible capacity decay as described in the following [15].

(1) $$V^{2+}+2{\mathrm{VO}}_{2}^{+}+2H^{+}\to 3{\mathrm{VO}}^{2+}+H_{2}O$$

(2) $$V^{2+}+{\mathrm{VO}}^{2+}+2H^{+}\to 2V^{3+}+H_{2}O$$

(3) $${\mathrm{VO}}_{2}^{+}+V^{3+}\to 2{\mathrm{VO}}^{2+}$$

(4) $${\mathrm{VO}}_{2}^{+}+2V^{2+}+4H^{+}\to 3V^{3+}+2H_{2}O$$

As shown in Equations (1)–(4), the crossover of vanadium ions through the membrane does not result in irreversible capacity decay, but rather self-discharges the electrolyte reversibly. However, a dissimilar rate of vanadium ion transport through the membrane reduces the state of charge window in one electrolyte and ultimately limits the cyclability and achievable total discharge energy [15].

Several parameters influence the vanadium ion and water crossover through the membrane, including electrolyte composition, reactor architecture, and operating conditions. The ion-exchange membrane attributes, including the polymer formulation, equivalent weight (EW), and degree of reinforcement, are tunable parameters affecting the mechanical stability as well as transport of the solute and the solvent [16]. A recent publication by the authors is dedicated to assessing the influence of membrane equivalent weight and reinforcement on the conductivity and selectivity of polymeric membranes [16]. This work, along with several others, suggests that the ionic conductivity of IEMs generally increases with increased dielectric constant of the membrane, solubility of charge balancing ion, and solvent concentration within membrane phase [17,18,19,20]. The ionic conductivity commonly decreases with increased EW and implementation of reinforcement. It has also been shown that the vanadium crossover decreases by increased EW and degree of reinforcement [20]. Therefore, there exists a trade-off between increased conductivity and electroactive ionic crossover within polymeric membranes. Several modeling and experimental studies have been dedicated to analyzing the vanadium ion transport through Nafion^®^ [15,21,22,23]. Therefore, transport parameters for vanadium ions through Nafion^®^ are relatively established in the literature, with some uncertainty remaining. However, the contributions from the surface barriers (electrode–membrane and membrane–membrane interface) to the charge and mass transport has not yet been extensively explored. Based on other literature, however, these interfaces are expected to play a significant role in net vanadium ion transport through the membrane [24].

In this work, a stack of IEMs has been utilized to assess the influence of surface barriers on the ionic conductivity and crossover for all-vanadium redox flow batteries. A unique test system equipped with UV/Vis spectroscopy was designed for measuring the ionic crossover rate in real time. An arrangement of commercially available Nafion^®^ membranes was analyzed for ionic conductivity as well as the vanadium crossover in the form of single and multiple membrane stacks. Contact impedance was measured, to prepare a selection guide for optimizing ionic conductivity and crossover of ionic species for all-vanadium redox flow batteries.

## 2. Materials and Method of Approach

### 2.1. Experimental Set-Up for Crossover Measurement

**Figure 1:**
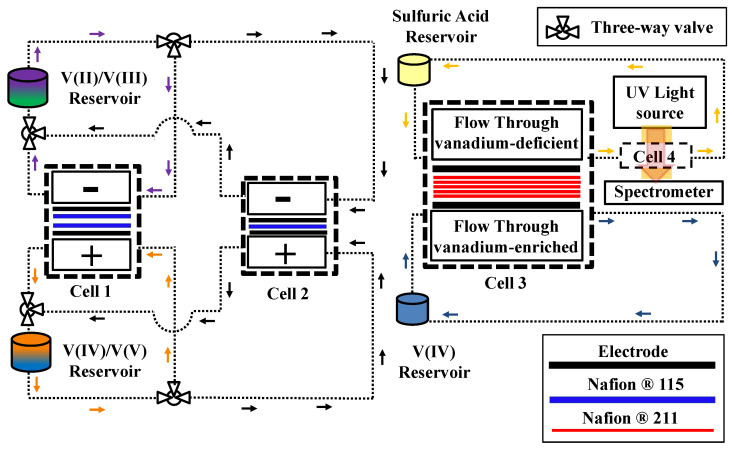
Test configuration used for measuring concentration-gradient-induced crossover; the setup includes several flow cells (Cell 1, 2, and 3), electrolyte reservoirs, and UV/Vis spectroscopy apparatus including UV light source, spectrometer, and UV cell (Cell 4) [16].

Figure 1 illustrates the experimental configuration used for measuring the ionic crossover through a stack of IEMs. A series of vanadium crossover experiments with commercially available ion-exchange membranes was conducted to assess the influence of surface barriers on the ionic crossover. To conduct crossover experiments, an in-house experimental test system (Figure 1) was utilized including flow cells, peristaltic pumps (Cole Parmer, Masterflex L/S, Vernon Hills, IL, USA), external reservoirs, light sources (Ocean-Optics, Dunedin, FL, USA), as well as ultraviolet/visible (UV/Vis) spectrometers (THORLABS, Newton, NJ, USA). For an operating cell, the concentration gradient and electric field are the major driving forces for vanadium crossover. The relative importance of electric-field-induced crossover over concentration-gradient-induced crossover flux has been already quantified for Nafion^®^ membranes in a recent publication from our lab [15]. Therefore, the focus here is only on concentration-gradient-induced crossover.

### 2.2. UV/Vis Spectroscopy

The cell architecture shown in Figure 1 (Cell 2) was used to explore the concentration-gradient-induced crossover for various combinations of the IEMs. As shown in Figure 1, the cell includes flow through flow plates (9 cm^2^), as-received carbon paper electrodes (39AA, one layer) along with a stack of various IEMs. Cell 1 was utilized to prepare the electrolytes. Cell 3 was a flow cell used for real-time monitoring the electrolyte. The UV/Vis spectroscopy including a light source, a spectrometer, and fiber cables, as illustrated in Figure 1, was used for assessing the concentration of vanadium ions diffused through the membrane stack. In Figure 1, Cell 1 and Cell 2 were used for preparing the electrolytes and conducting long term tests. Cell 1 had two Nafion^®^ membranes in its architecture for minimizing the crossover during bulk electrolysis.

Crossover experiments were executed with vanadium-enriched (1.5 mol/L VOSO_4_xH_2_O in 3.3 mol/L H_2_SO_4_) and vanadium-deficient (4.8 mol/L H_2_SO_4_) solutions separated by membranes. Previous studies [15,20] revealed that a sulfuric acid concentration of 4.8 mol/L on the vanadium-deficient side balances osmotic pressure across the membrane in Cell 3. Identical flow rates of 20 mL/min for both electrolytes were ensured by a two-channel peristaltic pump. After passing through Cell 3, both electrolytes flowed through UV/Vis flow-cells to quantify the composition of the vanadium-deficient side in real time. All crossover experiments were executed for 24 h with vanadium concentration in the deficient side measured each six hours according to a procedure explained in previous publications [15,20]. A similar experimental procedure was repeated for various configurations of the membranes.

### 2.3. Membrane Selection and Pretreatment

Commercially available perfluorinated Nafion^®^ membranes with various configurations (i.e., N117, N115, NR212, and NR211) were investigated. The N117 and N115 membranes are extruded polymers, whereas NR212 and NR211 are solution cast polymers. These cationic exchange membranes have the equivalent weight of 1100 g·mol^−1^ with nominal dry thickness of 177.8, 127, 50.8, and 25.4 µm for N117, N115, NR212, and NR211, respectively. The as-received membranes were soaked in a solution containing aqueous sulfuric acid (3.3 M) for more than a week at ambient temperature before testing.

### 2.4. Electrochemical Impedance Spectroscopy (EIS)

A Bio-Logic SP240 potentiostat (BioLogic Science Instruments, Seyssinet-Pariset, France) was used to conduct electrochemical impedance spectroscopy analysis. AC impedance spectroscopy (5 mV perturbation, BioLogic Science Instruments, Seyssinet-Pariset, France) was conducted at open circuit over a frequency range of 300 kHz to 100 mHz. In all measurements, the positive electrode served as the working electrode while the negative electrode was the counter electrode. The series resistance, assumed to be dominated by the membrane’s ionic resistance, was interpreted as the high frequency impedance where the imaginary component was zero.

### 2.5. Ionic Conductivity Assessment for Ion-Exchange Membranes and Electrolytes

**Figure 2:**
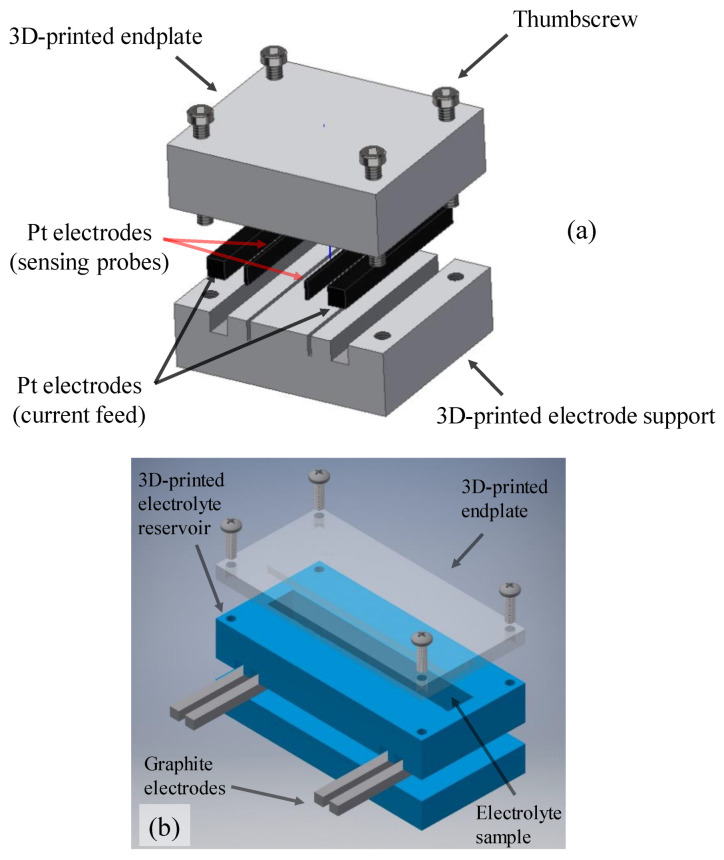
Schematic of the device designed in-house for measuring: (**a**) in-plane ionic conductivity of ion-exchange membranes (IEMs), (**b**) ionic conductivity of electrolytes [27].

To measure the in-plane ionic conductivity of the ion-exchange membranes, an in-house ionic conductivity cell was designed and fabricated (Figure 2a). As shown in Figure 2a, the conductivity cell enabled four-electrode AC impedance measurement of the IEMs. The ion-exchange membrane of interest was initially soaked in the solution at room temperature for more than a week; prior to the measurement, electrolyte droplets were gently removed from the surface. Subsequently, the membrane was placed between the top and bottom endplates of the conductivity cell (see Figure 2a); finally, the cell was assembled and the AC impedance measurement was conducted. Further details on the conductivity probe are available in a review article from our lab [6].

To measure the ionic conductivity of liquid electrolyte, an external conductivity meter is commonly used [25,26]. In this work, a unique conductivity cell was designed, and 3D printed in-house as shown in Figure 2b to probe the ionic conductivity of the solutions. The conductivity cell shown in Figure 2b was equipped with graphite electrodes providing a durable yet conductive medium in the highly acidic environment. The motivation for ex-situ conductivity measurement stems from the fact that in-situ measurement includes multiple contributions to a measured resistance. In this case, in-situ conductivity of the membrane has additional series resistances (contact and electronic) that cannot be disentangled by AC impedance spectroscopy. An additional benefit is that the ex-situ measurements can be executed more quickly than in-situ assessments.

To calculate the ionic in-plane conductivity of the membrane and electrolytes, the following equation was utilized [6].

(5) $$\sigma =\frac{L_{s}}{R_{s}S_{s}}$$

where $L_{s}$ is the distance between the voltage probes in the conductivity cells shown in Figure 2, $R_{s}$ is the high-frequency impedance of the sample (e.g., a membrane, electrolyte), and $S_{s}$ is the cross-sectional area. In our experiments, the impedance, $R_{s}$, was evaluated by measuring the high frequency (>50 kHz) resistance between the voltage probes.

A common obstacle facing ex-situ measurement is that the conditions are often dissimilar from those in the cell. Accurate ex-situ conductivity measurement was accomplished by soaking membranes in the electrolyte of interest for over a week prior to any measurement. It is noted here that conductivity measured in the manner presented here is the in-plane conductivity of the membrane; VRFBs are more strongly influenced by the through-plane conductivity. For membranes with isotropic properties, this distinction is irrelevant; however, for reinforced or otherwise anisotropic membranes, the in-plane conductivity can differ significantly from the through-plane conductivity. Often, the electrolyte–electrode–membrane boundary is a boundary condition for the membrane in both equilibrium and non-equilibrium conditions. It is evident in Equation (5) that sample geometry (thickness and width) strongly influences the calculated conductivity. Accurate measurement of these properties is difficult because ionic uptake induces elongation; in effect, the stress–strain characteristics of membranes is strongly dependent on the solutions in which they are soaked. Finally, the force applied in the conductivity cell must be consistent in order to ensure that multiple measurements have consistent contact between samples and electrodes. These issues have been addressed in this work via repeating the experimental procedure over various samples until repeatable measures were obtained. The average errors across all measurements were less than 5%.

## 3. Results

### 3.1. Assessing Membrane–Electrode Interface Impedance

**Figure 3:**
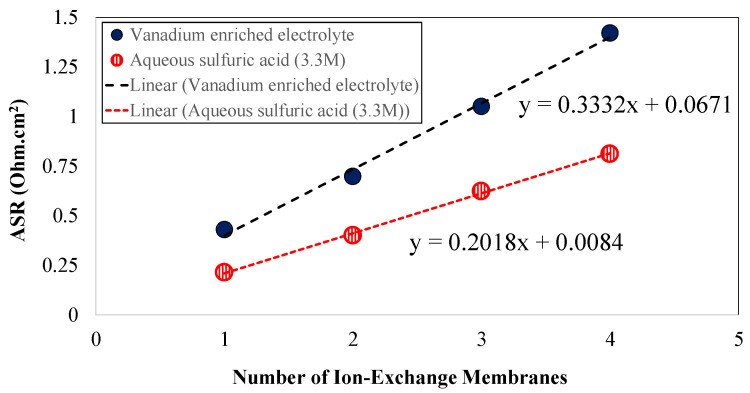
The area specific resistance (ASR) for all-vanadium redox flow batteries (VRFBs) assembled with multi-layer configurations of Nafion^®^ 115 (Note: The ASR values were corrected for the values without membrane).

To measure the resistance associated with the electrode–membrane interface, area specific resistance was assessed for a series of N115 membranes. Flow cells with 9 cm^2^ flow-through plates and as-received 39AA electrodes were used. A series of cells with one, two, three, and four layers of N115 ion-exchange membranes were assembled. In addition, area specific resistance was assessed for an identical cell architecture assembled with no membrane. This enabled quantification of the impedance associated with electrodes, electrolytes, flow plates, current collectors, and related contact resistances. Figure 3 includes the area-specific resistance associated with the VRFBs assembled with various vanadium redox flow battery configurations.

The no-membrane architecture included as-received carbon paper electrodes (one layer, 39 AA) along with flow-through flow plates and current collectors. The solutions of aqueous sulfuric acid (3.3 M) and enriched vanadium solutions (1.5M V(IV), 3.3M sulfuric acid) were circulated through the cell and the area specific resistance was assessed for this case. Similar experiments were repeated for cell architectures including one, two, three, and four layers of N115. The ASR associated with each case has been summarized in Table 1. In Table 1, the ASR values for the cells with membranes have been listed correcting from the ASR associated with the no-membrane architecture.

To quantify the impedance associated with the electrode–membrane interface, as shown in Figure 3, the ASR values have been plotted for different configuration of membranes with various flowing electrolytes. A linear trendline has been plotted for the data series and the *y*-axis intercept has been evaluated for the impedance associated with the electrode–membrane interface. As shown in Figure 3, for solution including aqueous sulfuric acid (3.3 M), the electrode–membrane impedance was ~0.008 ohm·cm^2^ and for the electrolyte including the enriched-vanadium solution (i.e., 1.5 M V(IV) and 3.3 M sulfuric acid), the interface impedance was 0.067 ohm·cm^2^. Therefore, the electrode–membrane interface resistance, accounting for the ionic sorption/desorption from both sides of the membrane–electrode interface, varies in the range of 0.008–0.067 ohm·cm^2^ for an operando all-vanadium redox flow battery with concentrated electrolytes.

### 3.2. Determining MacMullin Number for Nafion^®^ Membranes Used within VRFB Architecture

As illustrated in Figure 3, the ASR values corresponding to stacked IEMs were greatly influenced by the concentration of the flowing electrolyte. Considering the porosity of the membranes, it is important to quantify the dependency of membrane conductivity to the conductivity of the flowing electrolyte. The MacMullin number is an important index to explore the interplay between the ionic conductivity of the electrolyte and membrane. The MacMullin number for a porous separator can be formulated using the following [28]:

(6) $$N_{M}=\frac{\kappa_{\mathrm{electrolyte}}}{\kappa_{{\mathrm{mem}}_{\mathrm{eff}}}}$$

**Figure 4:**
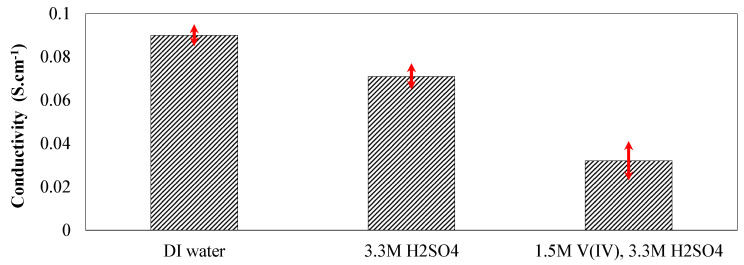
In-plane ionic conductivity of Nafion^®^ 115 equilibrated in different electrolytes.

As formulated in Equation (6), the MacMullin number ($N_{M}$) relates the conductivity of the electrolyte ($\kappa_{\mathrm{electrolyte}}$) to the effective conductivity of the porous membrane ($\kappa_{{\mathrm{mem}}_{\mathrm{eff}}}$). Therefore, to determine the MacMullin number, it is necessary to independently measure the conductivity of the electrolytes and membranes. In this work, conductivity cells (Figure 2) were used to measure the ionic conductivities. Several samples of Nafion^®^ 115 were used to assess the in-plane ionic conductivity and to confirm the repeatability of the measurements. Various bathing solutions including deionized (DI) water, aqueous sulfuric acid, and enriched vanadium solution were prepared, and the membranes were soaked in these bathing solutions for more than a week. Subsequently, the in-plane conductivities were evaluated using Equation (5). Figure 4 includes the in-plane conductivity of the Nafion^®^ 115 membranes soaked in different electrolytes. Similar analysis was conducted for assessing the in-plane ionic conductivity of NR211 membrane at similar testing conditions as shown in Figure S1 within Supplementary Information (SI). According to Figure 4, the in-plane conductivity of the Nafion^®^ membranes is a strong function of the bathing solution. The highest value of conductivity achieved for the membranes soaked in DI water (~0.09 S·cm^−1^) followed by aqueous sulfuric acid (~0.076 S·cm^−1^) and enriched vanadium solution (~0.032 S·cm^−1^).

The in-plane conductivity of the membranes soaked in aqueous sulfuric acid is significantly higher compared to enriched vanadium solution. The conductivity trend observed in this case is consistent with the ASR values shown in Figure 3 and Table 1.

**Figure 5:**
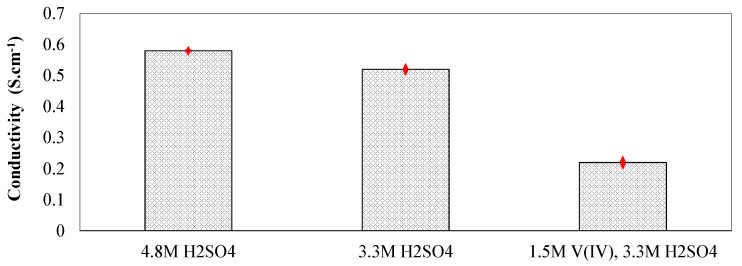
The ionic conductivity measured for various electrolytes.

To further assess the influence of electrolyte composition on the in-plane conductivity of the membranes, the ionic conductivity of the electrolytes was also measured ex-situ. Electrolytes with various configurations were prepared; i.e., aqueous sulfuric acid (3.3 M and 4.8 M) and enriched vanadium solution (1.5M V(IV), 3.3M sulfuric acid). The conductivity cell shown in Figure 2b was used to measure the ionic conductivity of the electrolytes. It is important to note that the conductivity measured for different electrolyte compositions was repeated across multiple cell configurations to confirm independent recording for the conductivities. Multiple configurations of conductivity cells with varied dimensions were designed and 3D printed in-house. Therefore, the ionic conductivities reported here were confirmed to be cell-independent. Figure 5 includes the ionic conductivities measured for various electrolyte compositions.

According to Figure 5, the ionic conductivity of the electrolytes varies significantly with the composition. The macroscopic description of the ionic conductivity can be formulated using the following equation [6,29].

(7) $$\kappa =F^{2}\underset{i}{\sum }z_{i}{}^{2}u_{i}c_{i}$$

In Equation (7), κ represents the ionic conductivity (S·cm^−1^), F the Faraday constant (C), $z_{i}$ charge of species *i*, $u_{i}$ mobility of ion *i* (cm^2^.V^−1^.s^−1^), and $c_{i}$ concentration of species *i* (mol·cm^−3^). Therefore, as formulated in Equation (7), the concentration of charge-carrying ions along with corresponding mobility are the major contributors to the ionic conductivity. As shown in Figure 5, increased acid concentration within the electrolytes increases the ionic conductivity in this range. However, enriched vanadium solution demonstrates significantly lower ionic conductivity. This trend contributes to the decreased ionic mobility of the major charge carriers (protons) within concentrated electrolyte solutions [30]. Decreased proton concentration upon adding VOSO_4_ to the aqueous solution containing sulfuric acid also contributes to the reduced ionic conductivity since the sulfate anions react with H^+^ ions to form bisulfate. Measuring ionic conductivity of the membranes along with the electrolytes enables quantifying the MacMullin number for Nafion^®^ perfluorinated membranes, as tabulated in Table 2.

As tabulated in Table 2, the MacMullin number for IEMs used within VRFB architectures with vanadium enriched and aqueous sulfuric acid solutions is in the range of 7.29–7.34. The error bars shown in Table 2 have been assessed based on the variation in experimentally measured in-plane ionic conductivity of the Nafion^®^ membrane soaked in different electrolytes. Therefore, for both electrolyte compositions (i.e., aqueous sulfuric acid and concentrated vanadium solutions), the ionic conductivity of the ion-exchange membranes is more than seven times lower compared to the conductivity of the electrolyte. Thus, it can be deduced that for aqueous electrolytes, the ionic conductivity of the electrolytes, regardless of the concentration of electroactive species, is not a limiting factor. As a result, to enhance the performance of aqueous RFBs with vanadium ions as the electroactive compounds, efforts must be dedicated to improving the ionic conductivity of the membranes.

In addition, according to Table 2, the variation in the MacMullin number as a function of electrolyte composition is inconsequential (~0.7%). Therefore, to further increase the ionic conductivity of the Nafion^®^ membranes, increasing the ionic conductivity of the electrolytes is an effective approach. A small increase in the MacMullin number for aqueous sulfuric acid solution (see Table 2) reveals that the uptake of vanadium co-ions within the Nafion^®^ structure affects the mobility of the protons in the membrane phase and adversely affect the conductivity. It is important to note that a higher MacMullin number for enriched vanadium solution is expected since the mobility of the charge carrying ions is further constrained in the membrane phase [20]. Although the influence of vanadium co-ion uptake is insignificant in the ionic conductivity, the transport of vanadium ions within the membrane phase is undesirable since it results in self-discharge, as discussed in the following section.

### 3.3. Formulating Vanadium Ion Crossover and ASR for a Stack of Ion-Exchange Membranes

As described in Section 3.1, stacking multiple layers of ion-exchange membranes results in increased ASR due to increased overall thickness and interfacial regions. Increased ASR increases the ohmic overpotential associated with the cell according to the following equation [6,31,32]:

(8) $$\eta_{ohmic}=\mathrm{ASR}.j$$

where $\eta_{ohmic}$ is the ohmic overpotential (V), ASR is the area specific resistance (ohm·cm^2^), and j is the current density (A·cm^−2^). As formulated in Equation (8), increased ASR directly increases the ohmic overpotential and subsequently reduces the power generated by the battery. In Section 3.2, it was shown that the conductivity of the Nafion^®^ membranes is directly correlated to the surrounding electrolyte. However, for the aqueous electrolytes with high concentration of sulfuric acid (2–5 M), the ionic conductivity of the electrolytes cannot be significantly altered. The other practical approach for reducing the ionic resistance imposed by the ion-exchange membrane is to reduce the membrane thickness. However, reduced membrane thickness results in increased crossover rate for a similar membrane microstructure. It has also been shown that via engineering membrane morphology, an optimum configuration can be achieved for reduced ionic crossover or reduced ASR without significantly altering the competing aspect [16].

**Figure 6:**
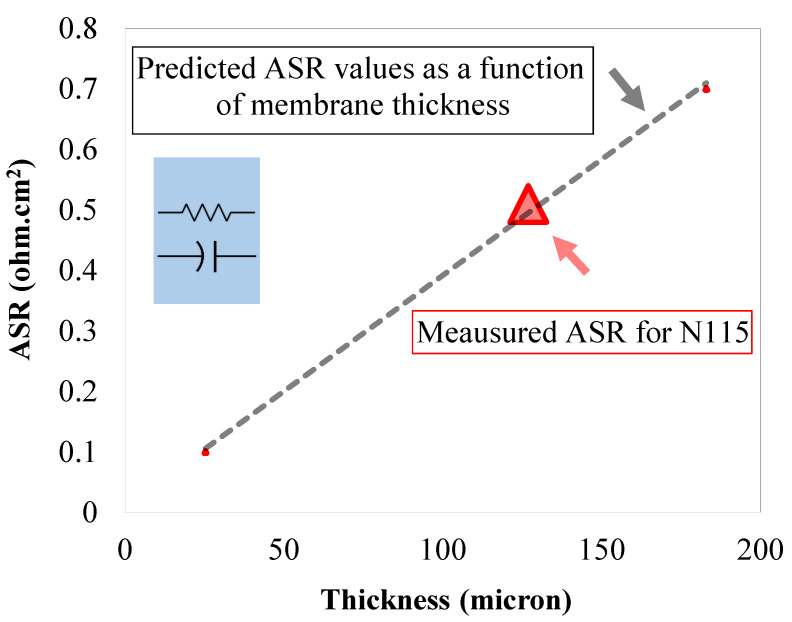
The ASR value for single-layer N115 and predicted ASR variation as a function of membrane thickness.

To investigate the influence of multiple ion-exchange membrane stacking on the ASR, the ASR for commercially available Nafion^®^ 115 (single layer) was initially measured. Figure 6 includes the ASR value measured for a single layer of N115 as a function of membrane nominal thickness (marked with a triangular symbol on Figure 6).

Within the acidic aqueous environment, the Nafion^®^ membrane is completely wetted by the electrolyte. Under this assumption, the ohmic resistance imposed by the membrane can be formulated using the following equation [28].

(9) $$R_{mem}=\frac{\tau_{m}l_{m}}{\varepsilon_{m}\kappa_{m}}$$

Here, $R_{mem}$ is the ohmic resistance associated with the membrane (ohm), $\tau_{m}$ is the tortuosity of the membrane, $l_{m}$ is the thickness of the membrane swelled in the electrolyte (cm), $\varepsilon_{m}$ is the porosity of the membrane, and $\kappa_{m}$ is the conductivity of the membrane (S.cm^−1^). In addition, for aqueous electrolytes, the dominant contributor to the ohmic overpotential is the ionic transport resistance imposed by the membrane; therefore, the area-specific resistance can be formulated as [6]

(10) $$\mathrm{ASR}=R_{mem}A_{m}$$

Here, $A_{m}$ is the projected area of the membrane (cm^2^). Therefore, combining Equation (9) and Equation (10), we can write:

(11) $$\mathrm{ASR}=\frac{A_{m}\tau_{m}l_{m}}{\varepsilon_{m}\kappa_{m}}$$

Therefore, as formulated in Equation (11), assuming the membrane properties remain unchanged; the ASR value is linearly dependent on the thickness of the membrane (for similar cell active areas). A similar trend has been shown in Figure 6 (dashed line). Therefore, a linear trend is expected for ASR based on the ASR value measured for N115.

**Figure 7:**
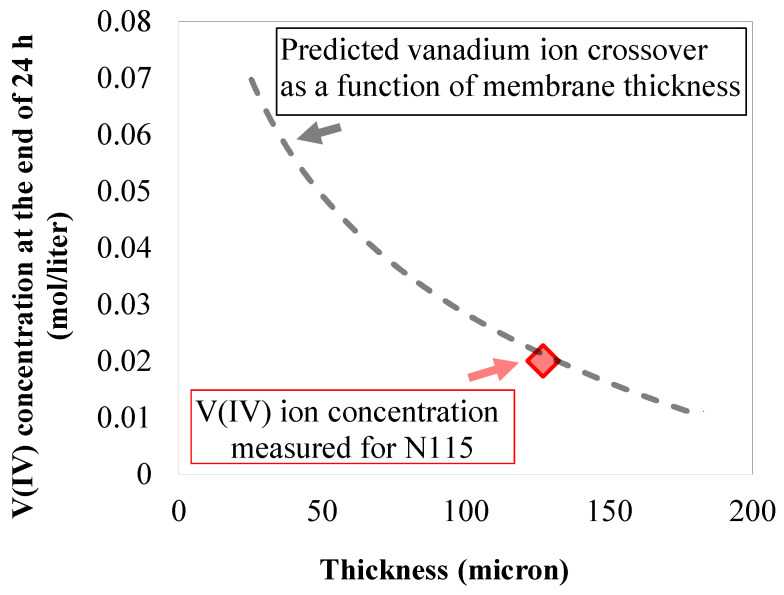
Concentration of the vanadium in the vanadium-deficient electrolyte at the end of the experiment for a cell with single-layer N115 membrane along with predicted vanadium crossover based on Fickian diffusion behavior.

To explore the influence of multiple membrane stacking on the crossover of electroactive compounds, the concentration-gradient-induced vanadium crossover was assessed for a single layer of N115. The schematic of the setup, along with experimental details, was already provided in Figure 1 and Section 2. Circulating vanadium enriched electrolyte (1.5M V(IV) and 3.3M sulfuric acid) in one side and vanadium-deficient electrolyte (4.8M sulfuric acid) in the other side of the reactor assembled with as-received carbon paper electrodes and a single layer Nafion^®^ 115 membrane, the total concentration of vanadium ions (V(IV)) was measured at the end of experiments (~24 h) within the membrane deficient electrolyte. Further details on the experimental procedure are available in a recent publication [20]. Figure 7 includes the concentration of vanadium within vanadium-deficient electrolyte at the end of experiment (highlighted in red). Assuming Fickian diffusion for concentration-gradient-induced crossover, the diffusive flux can be formulated in the form of Equation (12) [6]:

(12) $$J\left(x,t\right)=-D\frac{\partial C\left(x,t\right)}{\partial x}$$

The continuity equation in the membrane phase can be written in the form of Equation (13) assuming that the vanadium ions do not react in the membrane phase (zero source term in the continuity equation) [15].

(13) $$\frac{\partial C\left(x,t\right)}{\partial t}=-\nabla \cdot J\left(x,t\right)$$

Assuming 1D transport through the membrane thickness is also justified considering the dimensions of the ion-exchange membrane (through-plane-diffusion-pathway/in-plane-diffusion-pathway = ~0.004). Therefore, assuming Fickian transport in the membrane phase, the continuity equation can be written in the form of:

(14) $$\frac{\partial C\left(x,t\right)}{\partial t}=D\frac{\partial^{2}C\left(x,t\right)}{\partial x^{2}}$$

Considering a steady state solution, Equation (14) can be written in the form of:

(15) $$\frac{\partial^{2}C\left(x\right)}{\partial x^{2}}=0$$

It is important to note that the diffusive model based on Fickian behavior only accounts for the interaction of species (i.e., vanadium ions) with the solvent. More rigorous modeling approaches based on concentrated solution theories can be used for modeling solute interactions within the flux formulation [33]. However, the transport models based on concentrated solution theory require several transport parameters that have not yet been measured for all-vanadium redox flow batteries. Therefore, for an experimental assessment provided here, the Fickian-based transport model has been used since it does not significantly alter the major conclusions. Readers are encouraged to refer to other publications where more rigorous approaches have been implemented for modeling the flux of species [21,22,30]. Adopting a steady-state and 1D framework, the continuity equation formulated in Equation (15) can be written in the form of Equation (16):

(16) $$C\left(x\right)=C_{M/E}\left(1-\frac{x}{l_{m}}\right)$$

In Equation (16), $l_{m}$ is the length of the membrane swelled in the concentrated electrolyte as formulated in Equation (9). In addition, for deriving Equation (16), we have assumed that the diffused vanadium ions immediately react in the opposite electrolyte (See Equations (1)–(4)). In Equation (16), the maximum concentration within the membrane phase has been assumed to happen at the membrane–electrode interface ($C_{M/E}$). To explore the influence of membrane thickness on the crossover of vanadium ions, a transient solution for the continuity equation (Equation (14)) must be considered. Several prior efforts have focused on analyzing the transient solution. In the Supplementary Information (Equation (S1) through Equation (S27)), we have provided further details on deriving the characteristic time ($t^{*}$) as formulated in the following [31].

(17) $$t^{*}\approx \frac{l_{m}{}^{2}}{D}=F\left(l_{m}{}^{2}\right)$$

As formulated in Equation (17), for a similar membrane structure and internal morphology, the variations of characteristic time with the membrane thickness is second-order. Such a second-order trend has been shown schematically in Figure 7 as a function of membrane thickness. Consequently, the concentration-gradient-induced flux will approach zero (mathematically) as the membrane thickness approaches infinity.

The ionic transport formulation derived in Equation (15) strictly applies for the membrane phase. However, the concentration of vanadium ions measured with the setup illustrated in Figure 1 was based on the analysis of the vanadium ion concentration within the external electrolytes. Therefore, permeability of the ion-exchange membrane towards a particular electroactive ionic species (e.g., ϖ) can be formulated as

(18) $$P_{\varpi }=S_{\varpi }D_{\varpi }$$

In Equation (18), $P_{\varpi }$ is the permeability (mol·cm^−2^·min^−^^1^), $S_{\varpi }$ is the solubility, and $D_{\varpi }$ is diffusivity of species ϖ within the membrane phase (cm^2^·min^−1^). Equation (7) formulates the correlation between ionic conductivity and mobility of ionic species within the electrolyte. The Nernst–Einstein equation describes the mobility ($u_{\varpi }$) as a function of diffusivity for the ionic species [6].

(19) $$u_{\varpi }=\frac{qD_{\varpi }}{kT}$$

In Equation (19), q is the elementary charge ($q=1.6\times 10^{-19}\;C$), k is the Boltzmann constant, and T is the temperature [17]. Therefore, plugging Equation (19) in Equation (18), we derive:

(20) $$P_{\varpi }=\frac{S_{\varpi }u_{\varpi }kT}{q}$$

The membrane ionic uptake values for protons and vanadium ions (V(IV)) has already been measured for various concentrated bathing electrolytes [34]. The concentration of protons in the membrane phase decreases as a function of decreased concentration in the bathing solution. The same trend has also been observed for the vanadium V(IV) ions. Therefore, according to Equation (20), the solubility plays a key role in influencing the permeability of ionic species through the membrane.

The solubility within the membrane phase, being a strong function of concentration of ionic species ϖ in the adjacent electrolyte phase, can be manipulated via stacking multiple IEMs and this is the core reasoning for adopting this approach for reducing ionic crossover without significantly altering the ASR. In the following section, more details are provided.

### 3.4. Multilayer IEM Membranes for Reduced Crossover

To explore the influence of stacking multiple IEMs on the crossover of vanadium ions, the system shown in Figure 1 was utilized. Five layers of Nafion^®^ 211 were used along with flow-through flow plates and a single layer of as-received carbon paper electrodes (39AA, SGL, Germany). The concentration-gradient-induced crossover was explored based on the procedure explained in Section 2.

**Figure 8:**
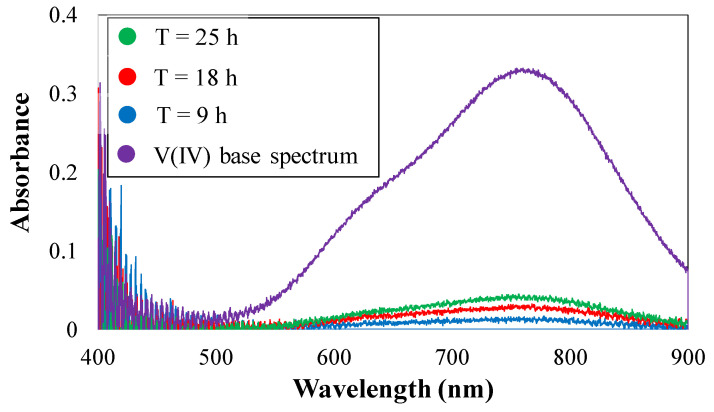
UV/Vis spectrum of vanadium-deficient electrolyte during crossover test for series of ion-exchange membranes (N115).

**Figure 9:**
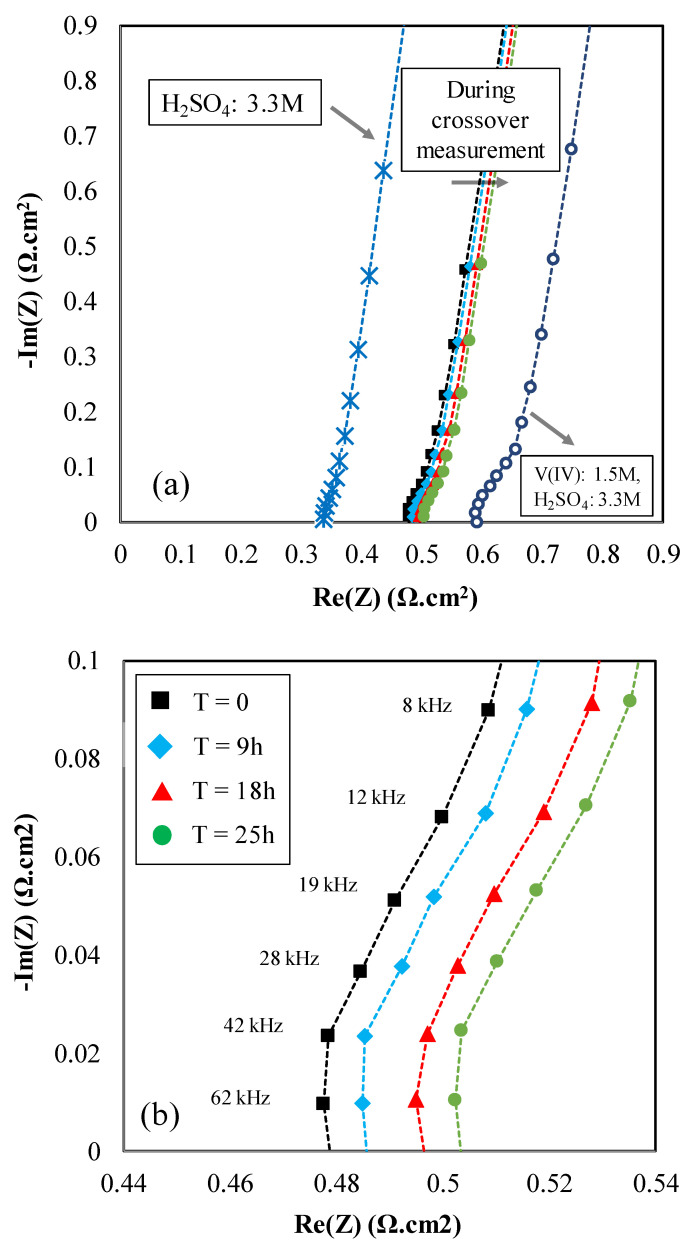
Electrochemical impedance spectroscopy for series of IEMs, (**a**) The spectra prior (contacting electrolyte at both sides: aqueous electrolyte with 3.3 M of H_2_SO_4_), during (vanadium-deficient and enriched electrolyte in different sides as described in the text), and after crossover measurement (contacting electrolyte: aqueous electrolyte with 1.5 M of V(IV), and 3.3 M of H_2_SO_4_ at both sides); (**b**) Spectra at high-frequency region.

The real-time UV/Vis spectra were recorded for vanadium-deficient electrolytes as shown in Figure 8. The spectra were analyzed using the scripts written in-house to obtain the concentration of vanadium ions in the vanadium-deficient electrolyte in real-time based on the procedure outlined in our previous publications [15,20]. As illustrated in Figure 8, the absorbance UV/Vis spectra at 760 nm was used to determine the concentration of vanadium ions within the vanadium-deficient electrolyte. Along with vanadium crossover measurement, electrochemical impedance spectroscopy was also utilized to assess the real-time influence of vanadium crossover on the area-specific resistance. Figure 9 includes the real-time EIS spectra recorded during the vanadium crossover experiment.

As shown in Figure 9a, the EIS spectra were obtained prior to the crossover test, where a similar aqueous sulfuric acid solution (3.3 M) was circulated in both sides of the redox flow battery. Subsequently, the vanadium deficient side was replaced with aqueous sulfuric acid with 4.8M acid concentration, and the vanadium-enriched side was set to 1.5M vanadium V(IV) and 3.3M sulfuric acid. The EIS spectra, as shown in Figure 9b, were recorded at t = 0, 9, 18, and 25 h into the crossover measurement. Finally, both solutions were replaced with vanadium-enriched solution and the EIS spectra were recorded.

As shown in Figure 9, the area-specific resistance for series of IEMs increases as a function of increased vanadium concentration in the vanadium-deficient electrolyte. This trend is consistent with the trend observed for single layer Nafion^®^ 115 (Table 1).

The increased ASR as a function of increased vanadium concentration in the vanadium-deficient electrolyte can be explained via exploring the MacMullin number calculated for the Nafion^®^ membranes (Table 2). As discussed in Section 3.2, the MacMullin number correlates the ionic conductivity of the ionic electrolytes contacting the membrane to the effective ionic conductivity of the membrane itself. Increased vanadium ion concentration in the vanadium-deficient electrolyte increases the MacMullin number due to increased membrane impedance; thus, the conductivity of the membrane does not scale linearly with the changes in conductivity of electrolytes when the electrolytes include vanadium ions.

**Figure 10:**
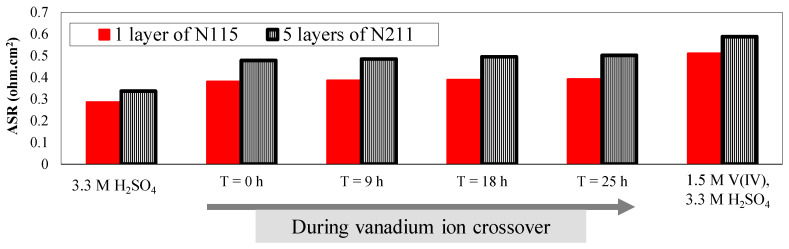
Comparison of ASR for multilayers of NR211 with a single layer N115 Nafion^®^ membrane during crossover measurement.

As demonstrated in Figure 5, conductivity of electrolytes including vanadium ions (V(IV)) is significantly lower compared to aqueous vanadium solutions (3.3 M acid concentration). Therefore, increased ASR as a function of increased vanadium concentration in the vanadium-deficient electrolytes is expected. To explore the influence of IEM stacking on the ASR, a comparison has been provided between five layers of Nafion^®^ 211 and a single layer N115 in Figure 10.

As shown in Figure 10, the ASR associated with multilayers of NR211 is higher compared to a single layer N115 regardless of the contacting electrolyte. Similar to single-layer N115, for the NR211 multilayer stack, increased vanadium ion concentration within the electrolyte-deficient electrolyte, increases ASR. However, to provide a real-time comparison in the cell level, the ohmic overpotential (Equation (5)) associated with each membrane configuration was assessed (see Table 3).

**Figure 11:**
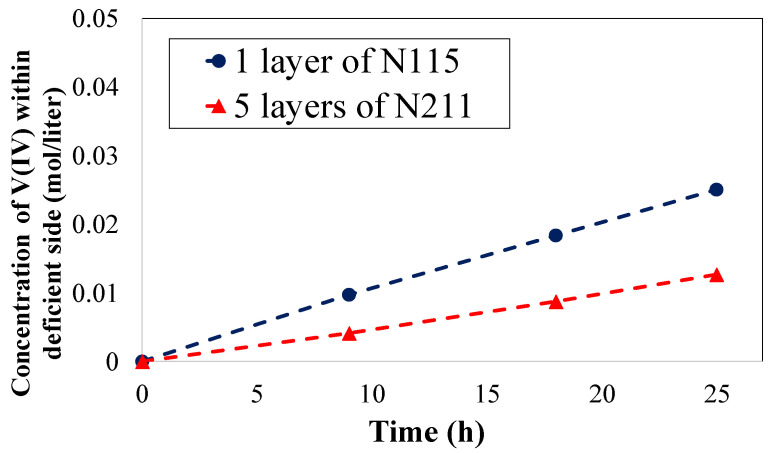
Comparison of concentration-gradient-induced crossover between 5 layers of NR211 and 1 layer of N115.

As tabulated in Table 3, the application of five layers of NR211 instead of a single-layer N115 membrane increases the ohmic overpotential from 51 mV to 59 mV at 100 mA·cm^−2^. For high performance VRFBs (i.e., high current density operation (e.g., 500 mA·cm^−2^)) the ohmic overpotential increases from 255 mV to 294 mV when a stack of NR211 membranes is replaced with a single-layer N115. Therefore, implementation of multilayer NR211 membranes, in comparison to one layer of N115, increases the ohmic overpotential by ~15%. In the Supplementary Information, we have also provided polarization curves along with voltage efficiency analysis for the VRFBs assembled with a single layer N115 versus NR211 membrane (see Figure S2). To have a more comprehensive picture, it is also necessary to explore the influence of IEM stacking on the crossover. A comparison of concentration-gradient-induced crossover between five layers of NR211 and one layer of N115 is provided in Figure 11.

**Figure 12:**
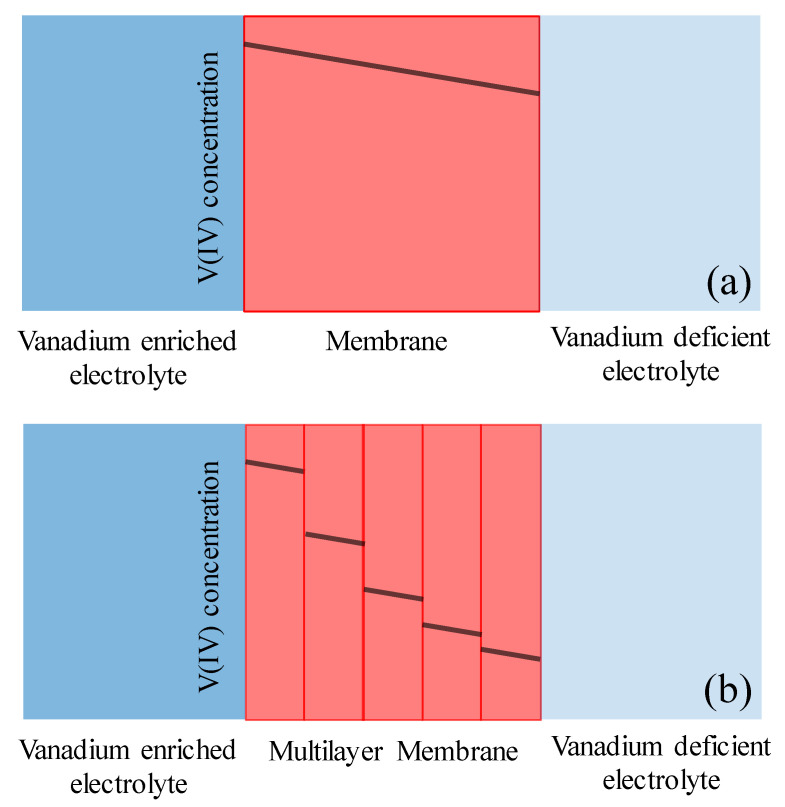
Schematic illustration of proposed reduction of ionic crossover for multilayers of IEMs, (**a**) 1 layer of N115, (**b**) 5 layers of NR211.

According to Figure 11, the concentration of vanadium ions in the vanadium-deficient side has been compared for both cases at various time frames (9, 18, and 25 h). As clearly illustrated in Figure 11, the implementation of multiple membrane stacking (in this case, five layers of NR211 instead of N115) significantly reduces the crossover of ionic species. For instance, comparing the concentration of vanadium ions diffused through the membrane to the vanadium-deficient electrolyte at the end of experiment (after 25 h of continuous operation under concentration-gradient), the vanadium crossover is reduced by 37% when five layers of NR211 are replaced with one layer of N115. The operando vanadium ion crossover measurement is consistent with the long duration cycling analysis performed for VRFBs assembled with a single layer N115 versus NR211 membrane (see Figure S3 within Supplementary Information). Therefore, the reduction in ionic species crossover compared to the increase in ASR is significantly higher when five layers of NR211 membranes are replaced with a single-layer N115 within the VRFB architecture. Figure 12 schematically illustrates the mechanism of reduced ionic crossover for multilayers of IEMs at steady state operation (see Equation (15)).

As schematically illustrated in Figure 12, imposing phase boundary within multilayers of IEMs results in reduced crossover since it results in ionic concentration discontinuity in the stack of membranes. It has already been shown that the ionic uptake from bathing solution reduces with reduced concentration of ions in the contacting electrolyte [34]. The ratio of vanadium concentration in the membrane phase to the adjacent electrolyte phase increases with decreased concentration of vanadium ions in the contacting solution [34]. Therefore, the concentration discontinuity in the membrane–membrane interface decreases as the concentration of vanadium ions in the adjacent membrane phase decreases. Such a discontinuity has been schematically illustrated in Figure 12.

It is also important to note that increased ASR observed for multilayers of IEMs is primarily due to contact resistances imposed via stacking multiple IEMs. Under the compression and due to variations in the adjacent electrolyte composition, IEMs considerably deform. The conductivity of ion-exchange membranes also varies when the composition of contacting electrolyte is altered. However, for ion-exchange membranes, the ionic conductivity is a strong function of water content; therefore, as long as an aqueous electrolyte wets the entire membrane stack, the increased ASR due to contact interface between multilayers of the IEM stack is not as substantial compared to the significant reduction in vanadium ion crossover.

Finally, it is critical to compare the variations of ASR and vanadium ion crossover across different cell architectures from the cost perspective, since it enables comparing these two metrics using a similar criterion. It is already been shown that the RFB price ($P_{0}$) per unit discharge energy ($E_{d}$) can be formulated considering the contributions to the overall cost from the reactor ($C_{Reactor}$), electrolyte ($C_{Electrolyte}$), balance-of-plant ($C_{BOP}$), and some additional sources ($C_{Additional}$) [35].

(21) $$\frac{P_{0}}{E_{d}}=C_{Reactor}+C_{Electrolyte}+C_{BOP}+C_{Additional}$$

Further details regarding various terms shown in Equation (21) are available elsewhere [35]. The variations in ASR and vanadium ion crossover directly affect the costs associated with the reactor and electrolyte shown in Equation (21).

Since all the other parameters affecting the reactor and electrolyte costs remain unchanged in Equation (21) when a single layer N115 is replaced with multilayer NR211, the reactor and electrolyte costs can be formulated as a function of ASR and ionic crossover [35].

(22) $$C_{Reactor}=\frac{R^{*}}{{ɸ}_{1}}$$

(23) $$C_{Electrolyte}=\frac{{ɸ}_{2}}{\varepsilon_{q,rt}}$$

Here, ${ɸ}_{1}$ and ${ɸ}_{2}$ are variables that remain unchanged when various membranes are used. Therefore, we have treated these parameters as constants in our formulation. Details regarding these parameters are available elsewhere [35]. In Equation (22), $R^{*}$ resembles the resistance of all the sources of overpotentials including ohmic ($R_{ohmic}$), kinetics ($R_{kinetics}$), and mass transport ($R_{MassX}$) resistances. As a result, it can be re-written in the form of:

(24) $$R^{*}=R_{ohmic}+R_{kinetics}+R_{MassX}$$

Replacing a single-layer N115 with multilayer NR211 directly influences the ohmic losses shown in Equation (24). Therefore, even if the most dominant overpotential within a cell architecture is the ohmic losses, the variation of reactor cost with ASR becomes linear (first-order). On the other hand, according to Equation (23), the round-trip coulombic efficiency ($\varepsilon_{q,rt}$) is a direct function of ionic crossover and shunt current in the cell stack. For a single cell configuration, the crossover of vanadium ions linearly influences the coulombic efficiency. Therefore, it can be concluded that the variations in ASR and vanadium crossover can be compared in terms of overall cost and both effects have a first-order influence on the cost, assuming the ohmic losses are the most dominant losses in a single-cell all-vanadium redox flow cell architecture. It is important to note that in our analysis, we assumed that the manufacturing costs associated with the membranes were not substantial and the overall material used for multilayer NR211 and single-layer N115 was comparable (similar thickness and identical polymer structure). Furthermore, in our cost analysis, we only focused on the reactor configuration and did not consider the costs associated with periodic re-balancing when the discharge capacity reaches a particular lower limit.

### 3.5. IEM Selection Criteria for Reducing Ionic Crossover in VRFBs

**Figure 13:**
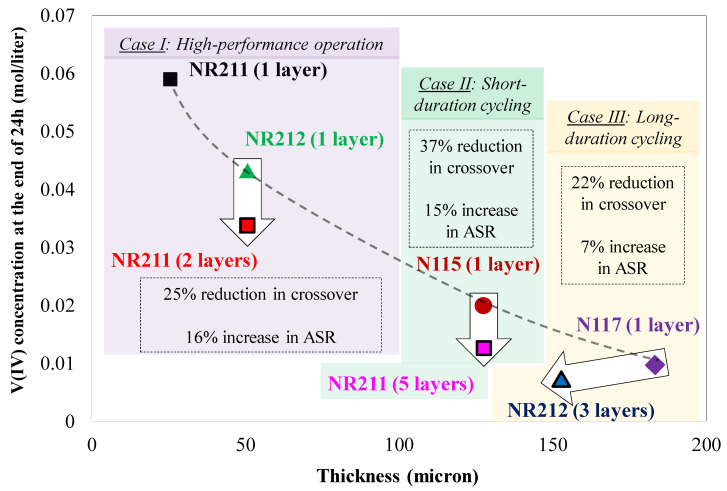
IEM selection chart for reducing ionic crossover in VRFBs. Dashed line represents an exponential fit to the experimentally measured vanadium ion (V(IV)) crossover data associated with the cells with single-layer Nafion^®^ membrane. The large white arrows demonstrate the improvement in capacity retention with multilayer thinner IEMs for different scenarios including high-performance operation as well as short- and long-duration cycling.

The comparison of multilayer NR211 with N115 in terms of ASR and concentration-gradient-induced crossover provided a promising approach for tacking crossover issues in VRFBs. Therefore, similar analysis was further expanded to cover other IEMs commonly used in the field. In this section (Figure 13), more comprehensive analysis has been provided including commercially available IEMs (N117, N115, NR212, and NR211).

Figure 13 includes an IEM selection chart for reducing ionic crossover in VRFBs. Three cases are included in Figure 13 which provide selection guidelines for designing the battery for a particular target. For high-performance VRFBs (Case I), it is necessary to minimize the ASR associated with the membrane. Therefore, among off-the-shelf IEMs, NR211 has the lowest ASR. However, the application of NR211 results in greater capacity decay due to crossover. Therefore, usually the second off-the-shelf choice is to utilize NR212. However, according to Figure 13, utilizing two layers of NR211 instead of NR212 only increases the ASR by 16%, but reduces ionic crossover by 25% compared to single-layer NR212. Case II is for designing VRFBs for relatively short-term capacity retention. Implementation of five layers of NR211 instead of single-layer N115 is recommended according to Figure 13 since the reduction in crossover (37%) is more pronounced compared to an increase in ASR (15%) when multilayer NR211s are utilized.

Designing VRFBs for extended and long-term duration is of special interest (Case III). Usually, Nafion^®^ 117 is the primary off-the-shelf choice. However, using three layers of NR212 instead of a single layer N117 results in 22% higher capacity retention and an increase in ASR is small (only ~7%). It is also important to note that if the costs associated with the membrane within VRFB systems scales up linearly with the nominal thickness of the membrane, the implementation of multilayer IEM stacking does not significantly alter the overall cost. In some cases (e.g., Case III), it even reduces the total cost, since the thickness of the membrane is reduced.

## 4. Summary and Conclusions

In this work, implantation of multilayer stacking of ion-exchange membranes for reduced ionic crossover was investigated. In particular, the trade-off between the ionic and mass transport impedance was evaluated across the membrane interface. Ex-situ conductivity cells were designed, built, and subsequently used to measure the in-plane conductivity of the membranes as well as the ionic conductivity of the contacting electrolytes. The MacMullin number for Nafion^®^ membranes was determined to correlate the ionic conductivity of the bathing solutions to the in-plane conductivity of the membranes.

A unique test system was utilized to assess the ASR and real-time crossover of vanadium ions. The ASR associated with electrode–membrane interface was also deduced. Subsequently, a series of off-the-shelf ion-exchange membranes (i.e., NR211, NR212, N115, and N117) were investigated in terms of ASR and vanadium crossover. Stacking multiple off-the-shelf membranes was explored as a promising yet inexpensive technique for tackling the rapid capacity decay issue associated with the operation of all-vanadium redox flow batteries. Design criteria for VRFBs utilizing stacks of off-the-shelf membranes were introduced for high-performance, short-term, and extended (long-duration) cycling experiments.

It was shown that the variations in ASR and ionic crossover can be compared in terms of the overall cost of the RFB systems. For example, it was shown that for long-term duration, implementing three layers of NR212 is preferable over a commonly used single-layer N117 since it results in 22% higher capacity retention with almost negligible variations in ASR. Therefore, for long-duration applications, the interfacial impedance can be engineered for passively reducing the ionic crossover and subsequently mitigating rapid capacity decay over extended cycling.

## Figures and Tables

**Table 1:** Area-specific resistance (ASR) for series of Nafion^®^ 115 membranes.

	ASR with Flowing Electrolyte Aqueous Sulfuric Acid (3.3M) (ohm·cm^2^)	ASR with Flowing Electrolyte Enriched Vanadium Solution (1.5M V(IV), and 3.3M Sulfuric Acid) (ohm·cm^2^)
No-membranes	0.07	0.08
1 layer of N115	0.21	0.43
2 layers of N115	0.40	0.70
3 layers of N115	0.60	1.05
4 layers of N115	0.81	1.42

**Table 2:** MacMullin number for IEMs within VRFBs configuration.

Electrolyte Type	MacMullin Number
Aqueous sulfuric acid (3.3M)	7.29 ± 0.01
Enriched vanadium solution (1.5M V(IV), and 3.3M sulfuric acid)	7.34 ± 0.02

**Table 3:** Comparison of ohmic overpotential for a series of NR211 versus single-layer N115.

	Ohmic Overpotential at 100 mA·cm^−2^ (mV)	Ohmic Overpotential at 500 mA·cm^−2^ (mV)
5 layers of NR211	59	294
1 layer of N115	51	255

## Supplementary Materials

Supplementary materials can be found at https://www.mdpi.com/2077-0375/10/6/126/s1. Figure S1: In-plane conductivity analysis for NR211 equilibrated in various bathing solutions. Figure S2. Performance analysis between cells assembled with 5 layers of NR211 vs. single-layer N115; (**a**) Polarization analysis; (**b**) Voltage efficiency. The electrolyte inclduded 1.5 M vanadium (state of charge: 50%) and 3.3 M sulfuric acid. Figure S3. Comparison of 5 layers NR211 with a sinlge-layer N115; (**a**) Discharge capacity decay; (**b**) Coulombic efficiency; (**c**) Capacity decay as a function of cycle number; (**d**) Capacity decay as a function of time. The cycling experiments were conducted at 100 mA·cm^2^ with voltage limits of 1.7 and 0.2 V. For polarization analysis as well as the long-duration cycling experiments, the electrolyte was 1.5 M vanadium (state of charge: 50%) and 3.3 M sulfuric acid. Throughout the experiment, the tempreature of the reactor and the storage tanks were cotrolled at 30 °C.
