# Supplementary material for: Direct Measurement of Crossover and Interfacial Resistance of Ion-Exchange Membranes in All-Vanadium Redox Flow Batteries
Source: Membranes (Basel). 2020 Jun 18;10(6):126. doi: 10.3390/membranes10060126 (PMC7345879; doi:10.3390/membranes10060126)
Supplement: Supplementary file 1 [file membranes-10-00126-s001.pdf]

membranes

Supplementary Information (SI) for:

Direct Measurement of Crossover and Interfacial Resistance of Ion-exchange Membranes in All-Vanadium Redox Flow Batteries

Yasser Ashraf Gandomi [1,2], Doug S. Aaron [1], Zachary B. Nolan [1], Arya Ahmadi [1] and Matthew M. Mench [1,3,*]

1 Department of Mechanical, Aerospace and Biomedical Engineering, University of Tennessee, Knoxville, Tennessee 37996, USA; ygandomi@mit.edu; daaron@utk.edu; znolan1@vols.utk.edu; znolan1@vols.utk.edu; mmench@utk.edu

2 Department of Chemical Engineering, Massachusetts Institute of Technology, Cambridge, Massachusetts 02139, USA; ygandomi@mit.edu

3 Energy and Transportation Science Division, Oak Ridge National Laboratory, Oak Ridge, Tennessee 37831, USA; mmench@utk.edu

* Correspondence: mmench@utk.edu; Tel.: +1-865-974-5115

1- In-plane ionic conductivity measurement of NR211 membrane soaked in various solutions

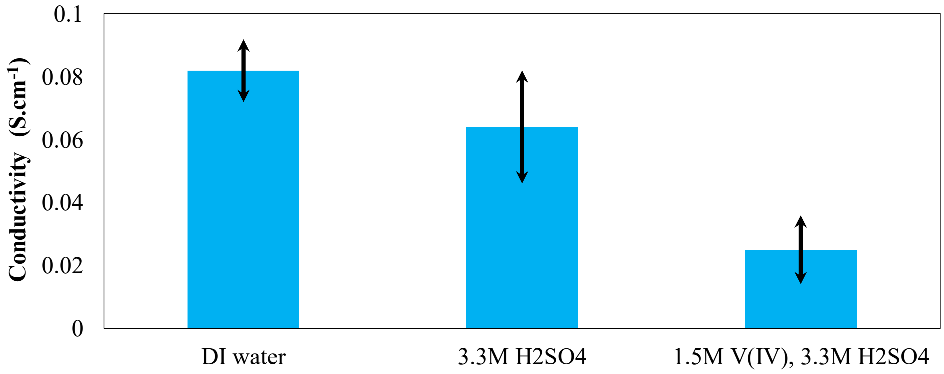

Figure S1. In-plane conductivity analysis for NR211 equilibrated in various bathing solutions.

2- Polarization analysis of all-vanadium redox flow batteries assembled with a single-layer N115 membrane vs. multi-layer NR211 membranes

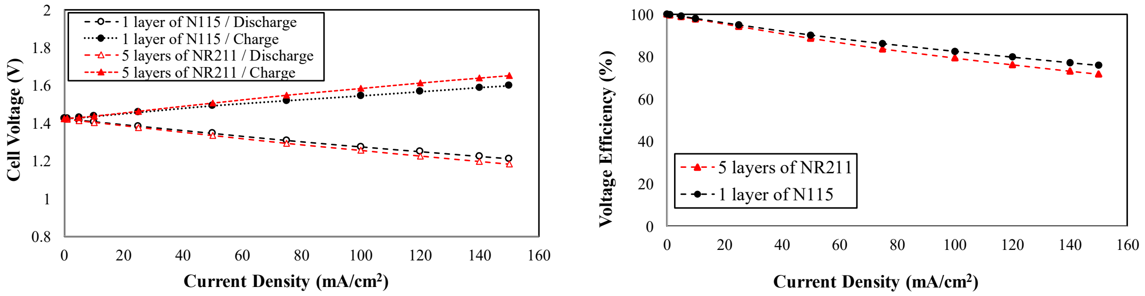

(a) (b)

Figure S2. Performance analysis between cells assembled with 5 layers of NR211 vs. single-layer N115; (**a**) Polarization analysis; (**b**) Voltage efficiency. The electrolyte inclduded 1.5 M vanadium (state of charge: 50%) and 3.3 M sulfuric acid. Throughout the experiment, the tempreature of the reactor and the storage tanks were cotrolled at 30 °C.

3. In-situ capacity fade analysis of all-vanadium redox flow batteries assembled with a single-layer N115 membrane vs. multi-layer NR211 membranes

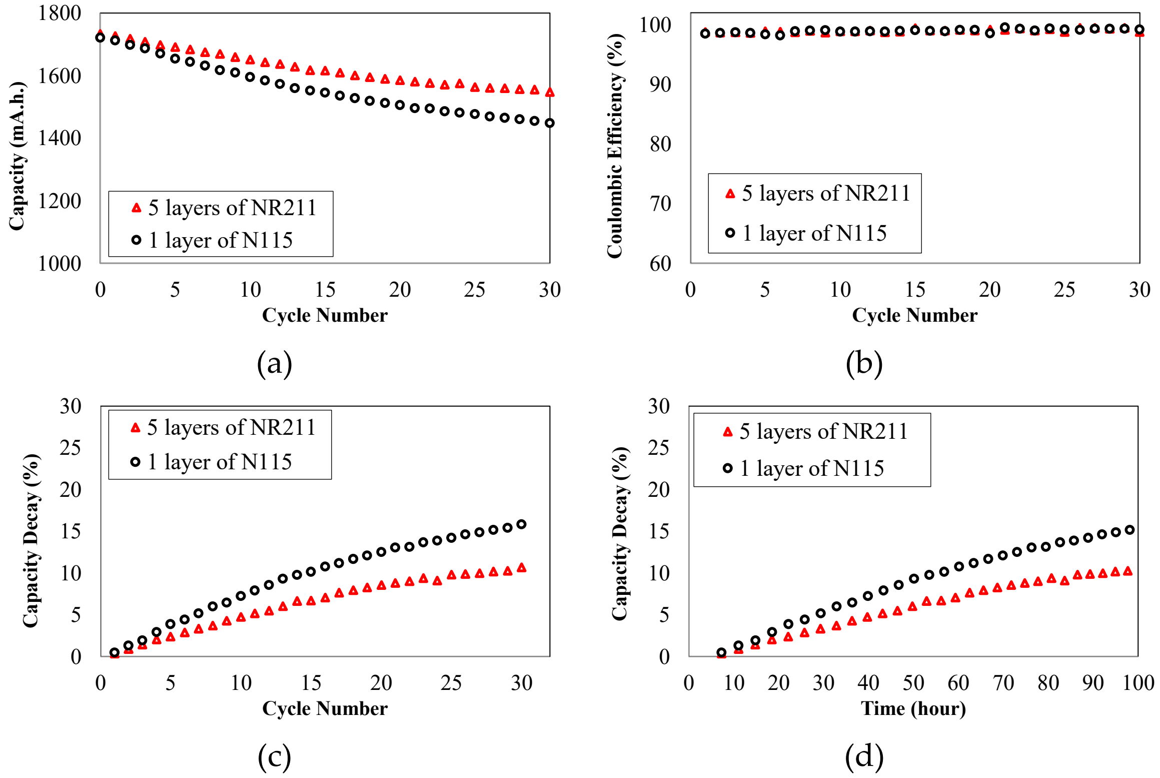

Figure S3. Comparison of 5 layers NR211 with a sinlge-layer N115; (**a**) Discharge capacity decay; (**b**) Coulombic efficiency; (**c**) Capacity decay as a function of cycle number; (**d**) Capacity decay as a function of time. The cycling experiments were conducted at 100 mA·cm^{-2} with voltage limits of 1.7 and 0.2 V. The electrolyte was 1.5 M vanadium (state of charge: 50%) and 3.3 M sulfuric acid. Throughout the experiment, the tempreature of the reactor and the storage tanks were cotrolled at 30 °C.

4- Analysis of transient (time-dependent) solution to the diffusion equation for the ionic transport within the membrane with Fickian flux formulation

Considering a 1-D diffusion, and assuming a Fickian flux, the conservation of mass can be written in the form of the following equation:

$$\frac{\partial C(x,t)}{\partial t} = D\frac{\partial^2 C(x,t)}{\partial x^2} \tag{S1}$$

For the steady state solution:

$$\frac{\partial^2 C(x)}{\partial x^2} = 0 \tag{S2}$$

The solution of the steady state becomes:

$$C(x) = ax + b \tag{S3}$$

Here, a and are b determined based on the boundary conditions. Refering to the thickness of the membrane swelled in the solvent as l_m; and assuming the following boundary conditions, we have:

$$C(x = 0) = C_0 \tag{S4}$$
$$C(x = l_m) = C_l \tag{S5}$$

Thus,

$$b = C_0 \tag{S6}$$
$$a = \frac{C_l - C_0}{l_m} \tag{S7}$$

Incorporating the boundary conditions, the steady state solution can be written in the form of:

$$C_{ss}(x) = (\frac{C_l - C_0}{l_m})x + C_0 \tag{S8}$$

For the transient solution, with the initial concentration of $C_i(x)$, the following form can be assumed:

$$\beta(x,t) = C(x,t) - C_{ss}(x) \tag{S9}$$

Here we have:

$$\beta(x,0) = C_i(x) - C_{ss}(x) = \beta_i(x) \tag{S10}$$

Here we assume that λ_m are the eigenfunctions, thus, the eigenvalues can be written as $-\delta_m^2$. Re-writing Equation (S1),

$$\frac{d^2\lambda_m(x)}{dx^2} = -\delta_m^2\lambda_m(x) \tag{S11}$$

Therefore, generic functions in the form of $\beta = A_m(t)\lambda_m$ satisfy Equation (s10) assuming that $A_m(t)$ also is the solution for Equation (S11).

$$\frac{dA_m(t)}{dt} = -D\delta_m^2 A_m(t) \tag{S12}$$

Solving Equation (S12), we derive:

$$A_m(t) = A_m(0)\mathrm{e}^{-D\delta_m^2 t} \tag{S13}$$

Therefore, the general from of the solution becomes:

$$\beta(x,t) = \sum_m A_m(t)\lambda_m(x) \tag{S14}$$

Separation of variables could also be used for deriving Equation (S14), here, we must also have:

$$\sum_m A_m(0)\lambda_m(x) = \beta(x,0) = \beta_i(x) \tag{S15}$$

Here, implementing the orthogonality attribute of λ_m, we derive:

$$\int_0^{l_m} \lambda_n^*(x)\lambda_m(x)dx = 0 \text{ (if } n \neq m) \tag{S16}$$

Or

$$\int_0^{l_m} \lambda_n^*(x)\lambda_m(x)dx = Y_n \text{ (if } n = m) \tag{S17}$$

Therefore, integrating Equation (S15) we derive:

$$A_n(0) = \frac{\int_0^{l_m} dx\lambda_n^*(x)\beta_i(x)}{Y_n} \tag{S18}$$

Combining Equation (S13) and Equation (S14):

$$\beta(x,t) = \sum_m \frac{\int_0^{l_m} dx \lambda_n^*(x)\beta_i(x)}{Y_m} \lambda_m(x) \mathrm{e}^{-D\delta_m^2 t} \tag{S19}$$

Imposing the boundary conditions, the solution can also be written in the form of:

$$\lambda_m(x) = Z_1 \cos\delta_m x + Z_2 \sin\delta_m x \tag{S20}$$

Therefore, on the boundaries:

$$0 = \delta_m(x = 0) = Z_1 \tag{S21}$$

$$0 = \delta_m(x = l_m) = Z_2 \sin\delta_m l_m \tag{S22}$$

Therefore, to satisfy Equation (S22), we must have:

$$\delta_m = \frac{m\pi}{l_m} \ (m \text{ is an integer}) \tag{S23}$$

Assuming ($Y_m = \frac{l_m}{2}$), the final solution can be simplified in the form of:

$$\beta(x,t) = \sum_m \frac{2}{l_m} \left(\int_0^{l_m} \sin\frac{m\pi x}{l_m} \beta_i(x) dx\right) \sin\frac{m\pi x}{l_m} \mathrm{e}^{-D\frac{m^2\pi^2}{l_m^2}t} \tag{S24}$$

Now, assuming $\beta_i(x) = \beta_i = \text{cte}$:

$$\int_0^{l_m} \sin\frac{m\pi x}{l_m} dx = 0 \ (\text{if } m \text{ is even}) \tag{S25}$$

$$\int_0^{l_m} \sin\frac{m\pi x}{l_m} dx = \frac{2l_m}{m\pi} \ (\text{if } m \text{ is odd } (2\mathrm{n}+1)) \tag{S26}$$

Therefore, the final form of the solution can be formulated as:

$$\beta(x,t) = \frac{4\beta_i}{\pi} \sum_{n=0}^{\infty} \frac{1}{2n+1} \sin\frac{(2n+1)\pi x}{l_m} \mathrm{e}^{-D\frac{(2n+1)^2\pi^2}{l_m^2}t} \tag{S27}$$

As formulated in Equation (s27), if $t \to \infty$, $\beta(x,t) \to 0$ with the characteristic time $t^* \approx \frac{l_m^2}{D}$.

References

1. Bard, A.J.; Faulkner, L.R.; Leddy, J.; Zoski, C.G. *Electrochemical methods: Fundamentals and applications*. wiley New York: 1980; Vol. 2.
2. Ashraf Gandomi, Y.; Aaron, D.; Houser, J.; Daugherty, M.; Clement, J.; Pezeshki, A.; Ertugrul, T.; Moseley, D.; Mench, M.M. Critical review—experimental diagnostics and material characterization techniques used on redox flow batteries. *Journal of The Electrochemical Society* **2018**, *165*, A970-A1010.
3. Newman, J.; Thomas-Alyea, K.E. *Electrochemical systems*. John Wiley & Sons: 2012.
4. Ashraf Gandomi, Y.; Aaron, D.; Mench, M.M. Coupled membrane transport parameters for ionic species in all-vanadium redox flow batteries. *Electrochimica Acta* **2016**, *218*, 174-190.
5. Ashraf Gandomi, Y.; Aaron, D.; Mench, M.M. Influence of membrane equivalent weight and reinforcement on ionic species crossover in all-vanadium redox flow batteries. *Membranes* **2017**, *7*, 29.
6. Ashraf Gandomi, Y.; Aaron, D.; Zawodzinski, T.; Mench, M.M. In situ potential distribution measurement and validated model for all-vanadium redox flow battery. *Journal of The Electrochemical Society* **2016**, *163*, A5188-A5201.
7. Ashraf Gandomi, Y.; Zawodzinski, T.A.; Mench, M.M. Concentrated solution model of transport in all vanadium redox flow battery membrane separator. *ECS Transactions* **2014**, *61*, 23-32.

© 2020 by the authors. Submitted for possible open access publication under the terms and conditions of the Creative Commons Attribution (CC BY) license (http://creativecommons.org/licenses/by/4.0/).
